# Introducing Cambridge Prisms: Global Mental Health

**DOI:** 10.1017/gmh.2022.62

**Published:** 2022-12-12

**Authors:** Judy Bass, Dixon Chibanda, Inge Petersen, Petr Winkler, Marit Sijbrandij, Rahul Shidhaye

**Affiliations:** 1 Global Mental Health, Johns Hopkins Bloomberg School of Public Health, Baltimore, MD, USA; 2 African Mental Health Research Initiative, University of Zimbabwe, Harare, Zimbabwe; 3 Centre for Global Mental Health, London School of Hygiene & Tropical Medicine, London, UK; 4 Centre for Rural Health, College of Health Sciences, University of KwaZulu-Natal, Durban, South Africa; 5 Institute of Global Health, University College London, London, UK; 6 WHO CC for Public Mental Health Research and Service Development (CZH-34), National Institute of Mental Health, Klecany, Czechia; 7 Clinical Psychology, VU University, Amsterdam, The Netherlands; 8 Psychiatry and Senior Research Scientist, Pravara Institute of Medical Sciences, Loni, India; 9 Clinical and Public Health Research, DBT-Wellcome Trust India Alliance, Hyderabad, India; 10 Care and Public Health Research Institute, Maastricht University, Maastricht, The Netherlands

**Keywords:** global mental health, global equity and inclusion, culture and context

## Abstract

With the launch of the Prisms Global Mental Health series, we are taking the opportunity to make explicit our vision for Global Mental Health. We strongly propose a Public Mental Health approach, incorporating culture and context and prioritizing equity and inclusion, particularly of previously marginalized groups. In using a Public Mental Health approach, we are framing Global Mental Health research as population-oriented research that seeks to understand the etiology, prevention, promotion, and treatment of mental and behavioral health problems with a strong emphasis on ‘knowledge generation’ which is relevant, transferable, and generalizable to different populations and settings. The public health approach also incorporates policy and systems research and evaluation, with a particular focus on accessibility and quality of care and human rights. By using the term Global, we are being explicit in acknowledging the role(s) of culture and context in all stages of research, from conceptualization through interpretation and dissemination. In centering equity and inclusion, we are advocating for a focus on populations who have been marginalized and have not been well represented within Global Mental Health research and active participation of voices of the populations that are included in the research. We are also working to promote participation of individuals from diverse and underrepresented communities and diverse experiences, including those with lived experience, in all stages of research pipeline: from conceptualization to publication of findings. Our readers will see these values and ideas operationalized in the choice of article topics and the published manuscripts as well as in the editorial and advisory board membership and selection of reviewers.

With the launch of the Prisms Global Mental Health series, we are taking the opportunity to make explicit our vision for Global Mental Health. We strongly propose a Public Mental Health approach, incorporating culture and context and prioritizing equity and inclusion, particularly of previously marginalized groups. In using a Public Mental Health approach, we are framing Global Mental Health research as population-oriented research that seeks to understand the etiology, prevention, promotion, and treatment of mental and behavioral health problems with a strong emphasis on ‘knowledge generation’ which is relevant, transferable, and generalizable to different populations and settings. The public health approach also incorporates policy and systems research and evaluation, with a particular focus on accessibility and quality of care and human rights. By using the term Global, we are being explicit in acknowledging the role(s) of culture and context in all stages of research, from conceptualization through interpretation and dissemination. In centering equity and inclusion, we are advocating for a focus on populations who have been marginalized and have not been well represented within Global Mental Health research and active participation of voices of the populations that are included in the research. We are also working to promote participation of individuals from diverse and underrepresented communities and diverse experiences, including those with lived experience, in all stages of research pipeline: from conceptualization to publication of findings. Our readers will see these values and ideas operationalized in the choice of article topics and the published manuscripts as well as in the editorial and advisory board membership and selection of reviewers.

We have selected a range of article topics to cover the length and the breadth of the field of Global Mental Health. We recognize, and promote, that many articles will fit within several topic categories, emphasizing the interdisciplinary nature of this field. These topics reflect our values through intentional acknowledgement of the role of culture and context, emphasis on diversity of experiences, and the validity and utility of results. The inclusion of the topic areas of Quality of Care and Human Rights is our explicit statement that these are relevant and key topics that must be considered in our field. Topics such as stigma, inclusion, recovery, and voices of people with lived experience of mental health conditions are crosscutting themes and principles that we would like to encourage authors to consider when building their articles.

**Table 1:** A brief description of each topic is provided below.

*Topic*	Description
Measurement	Psychometric properties, including validity, reliability, and accuracy of tools used in diverse settings, utility and application of diagnostic strategies, culturally appropriate translation of tools in multiple contexts
Etiology	Epidemiology, genetics, risk and protective factors (including social determinants of health)
Intervention	Efficacy, effectiveness, and cost-effectiveness of interventions, including individual. Group and community psychological, psychosocial and pharmacotherapeutic care, digital mental health, drug access/cost
Prevention	Early interventions, primary and secondary prevention; universal, selective, and indicated prevention
Promotion	Strategies to improve and increase mental health and well-being
Policy and Systems	Platforms of care (community-based care, integration in primary care, digital platforms), scale-up and sustainability, local and national mental health programs, international aid programs
Quality of Care	Patient-centered care, equitable treatment access and timely appropriate care, precision medicine
Human Rights	Community support and programs that address systemic rights abuses; local, national, and international policies
Teaching and Learning	Methods, competency assessment, expanding capacity, training non-mental health professionals

## Guiding perspectives of the co-editors in chief and senior editors

Judy Bass, PhD MPH, Professor of Global Mental Health, Johns Hopkins Bloomberg School of Public Health, Baltimore, Maryland, USA.

“My research career has been guided by the goal of addressing the ‘Who, What and Where’ questions of global mental health interventions and services: Who are the people whose mental and behavioral health needs are not being met; What are their needs and which interventions promise and/or effectiveness for meeting those needs; and Where (and How) should the interventions be implemented to be both accessible and appropriate.”

Dixon Chibanda MD, MPH, PhD Director African Mental Health Research Initiative, University of Zimbabwe, Associate Professor Centre for Global mental Health London School of Hygiene & Tropical Medicine.

“My main interest is to explore the intersection of indigenous knowledges and western models of care to develop sustainable interventions in global health”.

Inge Petersen, PhD, Research Professor, Centre for Rural Health, College of Health Sciences, University of KwaZulu-Natal, South Africa. Visiting Professor, Institute of Global Health, University College London.

“My research is driven by the need for social justice in the health system. To this end, I engage in collaborative embedded whole health systems research to optimize implementation of effective interventions along the care cascade. This with the view to reduce inequities and improve equal access to quality mental health services in resource-scarce settings”.

Petr Winkler, PhD, Director, National Institute of Mental Health, Czechia; Director, WHO CC for public mental health research and service development (CZH-34).

“My research is focused on improving mental health care in the region of central and eastern Europe; and it covers a wide range of public mental health topics, including psychiatric epidemiology, health service research, health economics, suicide prevention, and research on stigma and discrimination.”

Marit Sijbrandij, PhD, Professor in Clinical Psychology (chair: Trauma and adversities in global settings) VU University, Amsterdam, the Netherlands, Co-director of WHO CC for Research and Dissemination of Psychological Interventions.

“My main area of study evolves around the public mental health response to trauma and adversities. I am interested in developing and evaluating interventions to prevent mental health problems resulting from war and disaster, with a specific focus on refugee and migrant populations.”

Rahul Shidhaye, MD, PhD, Associate Professor of Psychiatry and Senior Research Scientist, Pravara Institute of Medical Sciences, Loni, India and DBT-Wellcome Trust India Alliance Intermediate Fellow in Clinical and Public Health Research. Visiting Researcher, Care and Public Health Research Institute, Maastricht University, The Netherlands.

“Translation of the ‘evidence’ into ‘practice’ to improve mental health outcomes in low-resource settings has been at the core of my research work. I am also deeply interested in understanding how interventions developed in non-European cultural contexts (e.g., Yoga-based interventions) can contribute to the field of Global Mental Health.”

